# Lifestyle intervention on psychotherapy and exercise and their effect on physical and psychological health in outpatients with schizophrenia spectrum disorders. A pragmatic clinical trial

**DOI:** 10.1192/j.eurpsy.2022.357

**Published:** 2022-09-01

**Authors:** B. Fernández-Abascal, P. Suárez-Pinilla, C. Cobo-Corrales, B. Crespo-Facorro, M. Suarez-Pinilla

**Affiliations:** 1 University Hospital Marqués de Valdecilla, IDIVAL Hospital, Department Of Psychiatry, Santander, Spain; 2 University Hospital Marqués de Valdecilla IDIVAL, Department Of Psychiatry, Santander, Spain; 3 University Cantabria, School Of Education, Santander, Spain; 4 University Hospital Virgen del Rocío - IBiS, Department Of Psychiatry,school Of Medicine, Sevilla, Spain; 5 University College of London, Department Of Neurodegenerative Disease, Institute Of Neurology, London, United Kingdom

**Keywords:** non-affective psychosis,, exercise, Quality of Life, cardiometabolic risk

## Abstract

**Introduction:**

Patients with Schizophrenia Spectrum Disorders (SSD) often lead unhealthy lifestyles with higher prevalence of obesity and unfavourable cardiometabolic parameters with less life expectancy and often worse quality of life compared with general population.

**Objectives:**

Evaluate the effectiveness of a combined intervention of exercise and psychoeducation in 48 SSD outpatients with metabolic syndrome (MetS), treated with second-generation antipsychotics and also aimed to explore if the effect persisted in a long-term follow-up of 24 months.

**Methods:**

The intervention included a 12-week aerobic exercise program and a session of lifestyle psychoeducation. Effectiveness was measured in terms a wide range of outcomes involving physical and psychological health, functioning, quality of life, physical activity and changes in motivation to exercise in the context of the self-determination theory.

**Results:**

The active intervention group showed benefits after Bonferroni correction over clinical global impression, identified motivation to exercise and changes of physical activity pattern. Maintenance of effects after 24 months of follow-up was observed for identified regulation to exercise and also for negative symptoms of psychosis (Table). Table. Effects assigned-group/time-over *p≤0.05 **p≤0.01.

**Table:** 

Variables	12weeks	24months	Time	Groupxtime	Groupx timex gender
	p	p	p	p	p
HDL (mg/dl)		0.021*			
Negative Syndrome Scale	0.044*	0.004**			
BREQ-2- Extrinsic regulation	0.008**	0.004**			
-External	0.026*				
-Introyected		0.038*			
-Identified	0.018*	0.015*	0.003**	0.002**	
BREQ-2-Intrinsic regulation				0.005**	0.004**
Pedometer (steps/day)			0.001**	0.006**

**Conclusions:**

A combined intervention on SSD outpatients with MetS showed effectiveness over several clinical parameters and functioning. Therefore, should be considered an essential part of the integral treatment in mental health services for SSD patients.

**Disclosure:**

No significant relationships.

